# A combination of upstream alleles involved in rice heading hastens natural long-day responses

**DOI:** 10.1007/s13258-024-01597-5

**Published:** 2024-11-20

**Authors:** Myung-Shin Kim, Joung Sug Kim, Sang Ik Song, Kyong Mi Jun, Su-Hyeon Shim, Jong-Seong Jeon, Tae-Ho Lee, Sang-Bok Lee, Gang-Seob Lee, Yeon-Ki Kim

**Affiliations:** 1 Department of Biosciences and Bioinformatics, Myongji University, 116 Myongji‑ro, Cheoin‑gu, Yongin, Gyeonggi‑do 17058 Republic of Korea; 2 Genomics Genetics Institute, GreenGene BioTech Inc., 16‑4 Dongbaekjungang‑ro 16beon‑gil, Giheung‑gu, Yongin, Gyeonggi‑do 17015 Republic of Korea; 3 Graduate School of Green-Bio Science and Crop Biotech Institute, Kyung Hee University, Yongin, Gyeonggi-do 17104 Republic of Korea; 4 Department of Agricultural Biotechnology, National Institute of Agricultural Sciences, Jeonju, 54875 Republic of Korea

**Keywords:** Rice, Photoperiod, Heading date, Recombinant isogenic lines, Single-nucleotide polymorphism

## Abstract

**Background:**

The female parental line Jinbuol (JBO, early heading) and two recombinant isogenic lines, JSRIL1 and JSRIL2, have been shown to flower 44, 34 and 16 days earlier, respectively, than the male parental line Samgwang (SG, late heading) in paddy fields.

**Objective:**

To explore how photoperiodicity-related genes are involved in differential heading among these lines.

**Methods:**

Deep sequencing was conducted for these lines, photoperiodicity-related genes (71) were categorized, and qRT-PCR was performed for some key genes.

**Results:**

Deep sequencing revealed a nearly even contribution of parental groups, with 48.5% and 45% of the chromosomes in JSRIL1 and JSRIL2, respectively, inherited from the female parent JBO; however, Chr6 contained the most biased parental contribution, with 99.4% inherited from the female parent. The variation in single-nucleotide polymorphisms (SNPs) among many known flower-inducing genes, including rice *GIGANTEA (OsGI); grain number, plant height* and *heading date 7 (Ghd7)*; and *EARLY HEADING DATE 1* (*Ehd1)*, was minimal. In the JSRILs, *HEADING DATE 1 (Hd1)* and *VERNALIZATION INSENSITIVE 3-LIKE 1 (OsVIL2) *originated from JBO, whereas *FLAVIN-BINDING, KELCH REPEAT, F BOX 1* *(OsFKF1)* originated from SG. Interestingly, *HEN1 suppressor 1 (OsHESO1)* originated from SG in JSRIL1 and JBO in JSRIL2. RNA sequencing and qRT‒PCR analyses of plants at the floral meristem stage revealed that transcriptional regulation through chromosomal restructuring and posttranscriptional regulation might control minute gene regulation, resulting in delayed heading in JSRILs.

**Conclusion:**

Our gene expression and SNP analyses of elite recombinant isogenic lines could be helpful in understanding how photoperiodicity-related genes in rice are modulated.

**Supplementary Information:**

The online version contains supplementary material available at 10.1007/s13258-024-01597-5.

## Introduction

Rice (*Oryza sativa*) was domesticated ∼8,200–13,500 years ago in the subtropical region of the Yangtze River in China. Recent demographic modeling using single-nucleotide polymorphism (SNP) data suggests a single-origin model featuring two main subspecies of Asian rice, indica and japonica, which were domesticated from the wild rice *Oryza rufipogon* and only recently separated into these subspecies (Molina et al. 2011). Domestication coincided with the expansion of the growth area from regions with a subtropical climate to various other regions. In particular, expansion into northern regions depended on the successful evolution of survival mechanisms by which reproduction is adjusted through the control of heading time to guarantee proper vegetative growth before the winter season begins (Izawa 2007). Thus, the heading date has been one of the main targets of research focused on understanding regional and seminal adaptations in rice cultivation.

Rice and *Arabidopsis* are considered short-day (SD) and long-day (LD) plants, respectively. After a certain period of vegetative growth, photoperiodic conditions are perceived by plants, and the signals are transmitted through multiple components. Forward and reverse genetics approaches have identified common evolutionarily conserved pathways involved in these processes; for example, the rice *GIGANTEA* (*OsGI)–Hd1–Heading date 3a (Hd3a)* pathway in rice is equivalent to the *Arabidopsis GI–CONSTANS (CO)–FLOWERING LOCUS T (FT)* pathway (Griffiths et al. 2003; Hayama et al. 2003). In addition, rice is a facultative SD plant in which heading is promoted under SD conditions and delayed under LD conditions, involving additional components. Florigens trigger genes involved in the shoot apical meristem (SAM)–to–floral apical meristem (FAM) transition. *Hd3a* and *RICE HEADING LOCUS T1* (*RFT1*) are the main florigens involved in SD and LD conditions, respectively (Komiya et al. 2008, 2009; Tsuji et al. 2011).

Photoperiodic pathways may be regulated through multiple controlling mechanisms, such as (post)transcriptional and (post)translational regulation (Zhou et al. 2021; Vicentini et al. 2023). The OsFKF1 protein contains LOV, F-box and Kelch-repeat domains and shares high amino acid identity with AtFKF1 (Nelson et al. 2000). The OsFKF1-OsGI interaction might regulate the blue light-mediated activation of *Ehd1* through physical interaction with CYCLING DOF FACTOR 1 (OsCDF1/OsDOF12; Han et al. 2015). OsFKF1 can also upregulate the expression of the floral activator *Ehd2* and downregulate the expression of the floral repressor *Ghd7* in the regulation of *Ehd1*. Mutation of *OsFKF1* delays heading under SD, LD and natural LD (NLC) conditions.

*Hd1* is a transcriptional activator and a key regulator of floral promotion under SD conditions (Putterill et al. 1995; Takahashi et al. 2009). In contrast, *Hd1* functions as an inhibitor under LD conditions (Lin et al. 2000; Yano et al. 2000). Under LD conditions, *Ehd1* is an important activator. *Ehd1*-anchored pathways typically involve *Ehd1, Ehd2, Ehd3, Ehd4, OsVIL2* and *MADS BOX GENE 51* (*OsMADS51*) (Doi et al. 2004; Matsubara et al. 2008, 2011; Gao et al. 2013; Wang et al. 2013; Jeong et al. 2016; Kim et al. 2007). Under LD conditions, additional components, such as *Heading date 2* (*Hd2/DTH7/OsPRR37*), *Hd5/DTH8, Ghd7, OsMADS51, OsVIL2,* and *LATE-FLOWERING 1* (*OsLFL1*), provide multilayer control in florigen production (Xue et al. 2008; Itoh et al. 2010; Wei et al. 2010; Koo et al. 2013;). Among these genes, *Ghd7, OsPRR37* and *Ehd1* are the main components of the molecular mechanism involved in critical day-length recognition (Itoh et al. 2010; Koo et al. 2013; Vicentini et al. 2023).

Chromatin restructuring through epigenetic regulation is also important in rice heading. This regulatory process has been elucidated through research on vernalization, in which heading is promoted in many plants after long-term exposure to the low temperatures of a typical winter. In *Arabidopsis*, *HEADING LOCUS C* (*FLC*), a heading repressor gene, is a major target of chromatin restructuring that puts the gene in a repressed state that is mitotically stable (Sung and Amasino 2004). VIL proteins are possible components of the polycomb repressive complex 2 (PRC2) complex that are involved in vernalization through histone modification, mostly deacetylation and methylation of downstream genes. Cold-induced expression of VIN3, a PLANT HOMEODOMAIN (PHD) finger-containing protein, is necessary for the formation of a histone deacetylase (HDAC) complex to deacetylate H3 in FLC chromatin. The complex of the DNA-binding protein VERNALIZATION 1 (VRN1) and the polycomb-group protein VRN2 can methylate H3 at Lys9 and Lys27 through the activation of an ENHANCER OF ZESTE [E(z)] homolog.

In rice, there are four VIL family members—*OsVIL1, OsVIL2, OsVIL3* and *OsVIL4*—all of which contain three conserved domains: PHD, FNIII, and VID (Zhao et al. 2010; Wang et al. 2013). OsVIL1 forms a PRC2-like complex to induce heading by suppressing *Oryza sativa late flowering* (*OsLF)* under SD conditions but delays heading by increasing *Ghd7* expression under LD conditions (Jeong et al. 2016). LEAF INCLINATION 2 (LC2, OsVIL3) and OsVIL2 (possible components of the PRC2 complex) promote rice heading. LC2 binds to the promoter region of the floral repressor OsLF and represses its expression via histone H3 lysine 27 (H3K27) trimethylation (H3K27me3). OsLF directly regulates *Hd1* expression through binding to the *Hd1* promoter (Wang et al. 2013). Insertion mutations in *OsVIL2* caused late heading under both LD and SD conditions (Yang et al. 2013). OsVIL2 induces heading by repressing *O. sativa LEAFY COTYLEDON 2 and FUSCA 3-LIKE 1* (OsLFL1).

The rice E(z) genes *SET DOMAIN GROUP 711* (*SDG711)* and *SDG718*, which encode the key PRC2 subunit that is required for H3K27me3, are involved in LD and SD regulation of key heading genes, respectively. *SDG711* is induced under LD conditions and represses *OsLF* and E*hd1*, leading to late heading. *SDG718* is induced under SD conditions and represses *OsLF*, a repressor of *Hd1* (Zhao et al. 2011), leading to increased expression of *Hd1*, which activates Hd3a and causes early heading (Liu et al. 2014). The SET PROTEIN 33 (OsSET33/SDG723/OsTrx1) protein is recruited to the promoter of *Ehd1* through interaction with OsSET33/SDG723/OsTrx1 interaction protein 1 (SIP1). The SET domain at the C-terminal end of OsTrx1 has histone H3 methyltransferase activity, leading to changes in chromatin structure. Mutations in *OsTrx1* cause late heading under LD conditions (Choi et al. 2014). Mutations in SIP1 result in a late heading date under LD and SD conditions (Jiang et al. 2018). Defects in *OsTrx1* or *SIP1* lead to reduced H3K4me3 levels at *Ehd1*, thus reducing *Ehd1* expression.

Posttranscriptional processing through microRNAs (miRNAs), small interfering RNAs (siRNAs), and piwi-interacting RNAs (piRNAs) is also important in controlling the photoperiod. In *Arabidopsis*, the turnover of these small RNAs is controlled by the methyltransferase HUA ENHANCER 1 (HEN1), which involves 2′ O-methylation of the 3′ terminal ribose (Chen et al. 2002), and HEN1 SUPPRESSOR 1 (HESO1), which involves 3′ uridylation, the addition of nontemplated nucleotides, predominantly uridine, generated through 3′-to-5′ truncation in small RNA (Zhao et al. 2012). HESO1 functions as a suppressor of the *hen1* mutation and results in pleiotropic phenotypes. *OsHESO1* is a rice homolog of *Arabidopsis thaliana HESO1*. A genome-wide association study (GWAS) of 176 whole-genome sequences revealed that rice plants with haplotype B presented a later heading date (Yano et al. 2016).

We developed 400 recombinant isogenic lines (RILs) via *Oryza sativa* spp. japonica cv. Jinbuol (JBO, Oumochi296/Isikaris//Kamaikumochi38, Jinbu11) as the female parent and cv. Samgwang (SG, Suwon361/Hwayeong, Suwon474) as the male parent. JBO and 2 RILs, JSRIL1 and JSRIL2, show heading 44, 34, and 16 days earlier, respectively, than SG does in Jeonju, Korea (35.83 N 127.05 E). An analysis of the genome-wide SNPs among the parents and JSRILs revealed that the parents have similar contributions overall, but parental biases are observed at many chromosomal levels, especially in photoperiodicity-related genes. SNP analysis of photoperiodic genes among these RILs revealed the following: no SNPs were detected in 63 genes, including *OsGI, Ghd7* and *Ehd1*; maternal JBO SNPs were detected in 5 genes, *Hd1, Hd3a, OsVIL2, SDG711*, and *CRYPTOCHROME 1B* (*OsCRY1b)*; paternal SG SNPs were detected in 2 genes, *SDG723* and *OsFKF1*; and a reversal in terms of heading days in was detected in 1 gene, *OsHESO1*. These results might explain the shortened or delayed flowering of the RILs compared with those of the parental lines. Transcriptome and qRT‒PCR analyses confirmed the characteristics of the photoperiodic network of these groups. Gene expression is regulated through transcriptional and posttranscriptional processes involving chromosomal restructuring, such as (de)acetylation, (de)methylation and (post)transcription.

## Materials and methods

### Plant materials

Approximately 400 RILs were generated using JBO as the female parent and SG as the male parent. JBO headed on Jul. 12, whereas SG headed on Aug. 15, in a paddy field at the National Institute of Agricultural Sciences in Jeonju, Korea (Kim et al. 2021). SG has high seed quality and has been commercialized in Korea. These RILs have currently produced more than 8 generations, and traits related to panicle development and various yield factors have been documented. Seeds were germinated in a greenhouse on May 8 and transplanted into the paddy field. Phenotypic observations and measurements were taken from plants growing in paddy fields. Fifteen to twenty plants were observed for heading. A one-tailed t test was performed for heading days.

### Genome sequencing

The sequencing library was prepared by random fragmentation of the DNA, followed by 5’ and 3’ adapter ligation. Adapter-ligated fragments were then amplified via PCR and gel purified. Illumina SBS technology utilizes a proprietary reversible terminator-based method that detects single bases as they are incorporated into DNA template strands. The raw sequencing data generated with the Illumina HiSeq 4000 platform (Illumina.com) were converted into raw data for further analysis. Approximately 14 Gb (40 × genome) of sequence data were obtained for the parents and the 2 RILs (Table S2). The additional sequencing data of the Nipponbare1 (SRR1043564), IndicaHR12 (SRR3056468), and Kitaake (SRR7789808) accessions were downloaded from the NCBI Sequence Read Archive (SRA) database. Kasalath data in vcf format were downloaded from the OryzaGenome database (http://viewer.shigen.info/).

### SNP and indel calling and phylogenetic analysis

The sequencing reads were trimmed with sickle v1.33 (https://github.com/najoshi/sickle) to generate high-quality paired-end files. Read mapping and variant calling were performed with GATK best practices (McKenna et al. 2010). Briefly, trimmed reads were mapped to the rice (*O. sativa* Nipponbare) reference genome sequence (IRGSP-1.0_genome) obtained from the RAP database (Sakai et al. 2013) via Bowtie 2 with default parameters (Langmead and Salzberg 2012). SAMtools v1.2 (Danecek et al. 2021) was used to index and sort the BAM and SAM files. Duplicate reads were filtered, and all reads were assigned to a read group in the output BAM file with Picard Tools (broadinstitute.github.io/picard/). The local realignment of reads around indels and variant calling were performed via GATK v3.5 (DePristo et al. 2011). Initially, 1,114,912 SNPs and 86,451 indels were detected. Quality filtering of initial SNPs was performed via VariantFiltration and SelectVariants implemented in GATK v3.5 according to the following criteria: ReadPosRankSum of <  − 8.0, MQRankSum <  − 12.5, QUAL < 30.0, QD < 3.0, FS > 06.0, mapping quality (MQ) < 40.0, genotype-filter-expression DP < 5, and genotype-filter-expression GQ < 20.0, select-type-to-include SNP, restrict-alleles-to BIALLELIC. VCFtools v0.1.16 (Danecek et al. 2021) was used to add the Kasalath vcf (vcf-merge) and remove the loci containing missing genotypes (–max-missing 1.0). Before the missing data were filtered out, the missing genotypes from Kasalath were converted to 0/0.

The sorted sequence variations and their effects on variants were analyzed with SnpEff 4.3i (https://pcingola.github.io/SnpEff/) from a vcf file. Phylogenetic analysis was performed with SNPhylo v.20140701 (Lee et al. 2014). A vcf file containing the data of JBO, JSRILs, SG, Nipponbare1, Kitaake, and Kasalath was used for the analysis. In addition, in an extended analysis with KRICE_CORE (Kim et al. 2016), the SNP data were manually merged via a Perl script, resulting in a list of common SNPs (468,994). Correlation information was generated in Newick format and transformed into a network, and circular tree diagrams were generated via the Molecular Evolutionary Genetics Analysis program with the toggle scale option (MEGA XI; Tamura et al. 2021).

### Calculation of parental contributions to the RILs

To calculate parental allelic segregation in the two RILs (JSRIL1 and JSRIL2), filtered SNPs were converted to ABH genotypes via the GenosToABHPlugin implemented in Tassel v5.2.89 (ref). JBO and SG were selected as parents A and B, respectively. The ABHgenotypeR package (Furuta et al. 2017) in R was used to calculate and visualize parental allelic segregation.

### RNA-seq analysis

Total RNA was extracted from single rice tillers of JBO, SG, JSRIL1, and JSRIL2 plants collected before heading, at midnight on June 22, June 23, July 6, July 13, and July 20 via the Hybrid-R system (GeneAllk, Republic of Korea). Total RNA was also extracted from the SG line on May 19 (17 DAS) as a reference. cDNA was synthesized from 1 µg of total RNA via the TruSeq Stranded mRNA Library Prep Kit (Illumina, USA). The fragmented cDNA was then purified, amplified via PCR and sequenced with a NovaSeq 6000 device. For each sample, 6.0–8.0 Gb (64–88 × 10^6^ paired-end reads) were generated. The raw sequence reads were mapped to the rice genome sequence (IRGSP-1.0_genome) in the Rice Annotation Project (RAP) database (http://rapdb.lab.nig.ac.jp). HISAT2 and SAMtools were used to process sequence reads (Kim et al. 2019; Li et al. 2009). Exon counts were obtained with dexseq_count.py in the DEXSeq package (Anders et al. 2012). Differential exon usage was tested with the DEXseq and limma packages in Bioconductor (Ritchie et al. 2015). The median count was adjusted by adding 1 to avoid trivial division errors. Significant transcripts were selected according to the following criteria: a log2-based ratio greater than abs(1.0) associated with adjusted p values less than 0.05 and a count greater than 30. Gene Ontology (https://geneontology.org/) and enrichment analyses were performed with Pantherdb (https://pantherdb.org/) via pthr_go_annots.py (https://github.com/pantherdb/pantherapi-pyclient). The adjusted P values of the GO enrichment data were scaled from 0 to 5 or  − 5 for the up- and downregulated genes, respectively, as shown in Table S6. For further analysis of significant transcripts from RNA-seq counts and GO terms, the distance and cluster were calculated with the dist function associated with the Euclidean method, and hierarchical clustering was performed with the hclust function with parameters, with an average based on the R-language. The enriched downregulated and upregulated genes were color coded with green and red, respectively, in the heatmap.2 function.

### Quantitative real-time PCR

For qRT‒PCR, 1 µg of total RNA was subsequently used as a template for reverse transcriptase reactions via a RevertAid H Minus First-Strand cDNA Synthesis Kit (Fermentas, USA) according to the manufacturer’s instructions. PCR was performed with one-hundredth of the first-strand cDNA mixture and gene-specific primers. The PCR conditions used were as follows: 10 min at 94 °C and 30 cycles of 30 s at 94 °C, 30 s at 55 °C, and 30 s at 72 °C. The 20 μL reaction volume contained 10 μL of Solg™ 2X Real-Time PCR Smart mix [Solgent, Korea], 2 μL of cDNA, 1 μL of primers, and 7 μL of water, and real-time PCR was performed via a CFX96 touch real-time PCR detection system (Bio-Rad, USA) with three technical replicates. Expression was assessed by evaluating threshold cycle (CT) values. The relative expression levels of the tested genes were normalized to the transcript level of *UBQ5* and calculated via the 2^−ΔΔ^CT method (Livak and Schmittgen 2001). Gene-specific primers were designed via the Primer-BLAST tool (http://ncbi.nlm.nih.gov) and are listed in Table S7. The data were summarized with Perl script (https://www.perl.org), and one-way analysis of variance (ANOVA) and significance tests were performed with the DunnettTest function in the DescTools library in R statistical language (https://cran.r-project.org/).

## Results

### Variations in heading date among RILs

**Fig. 1:**
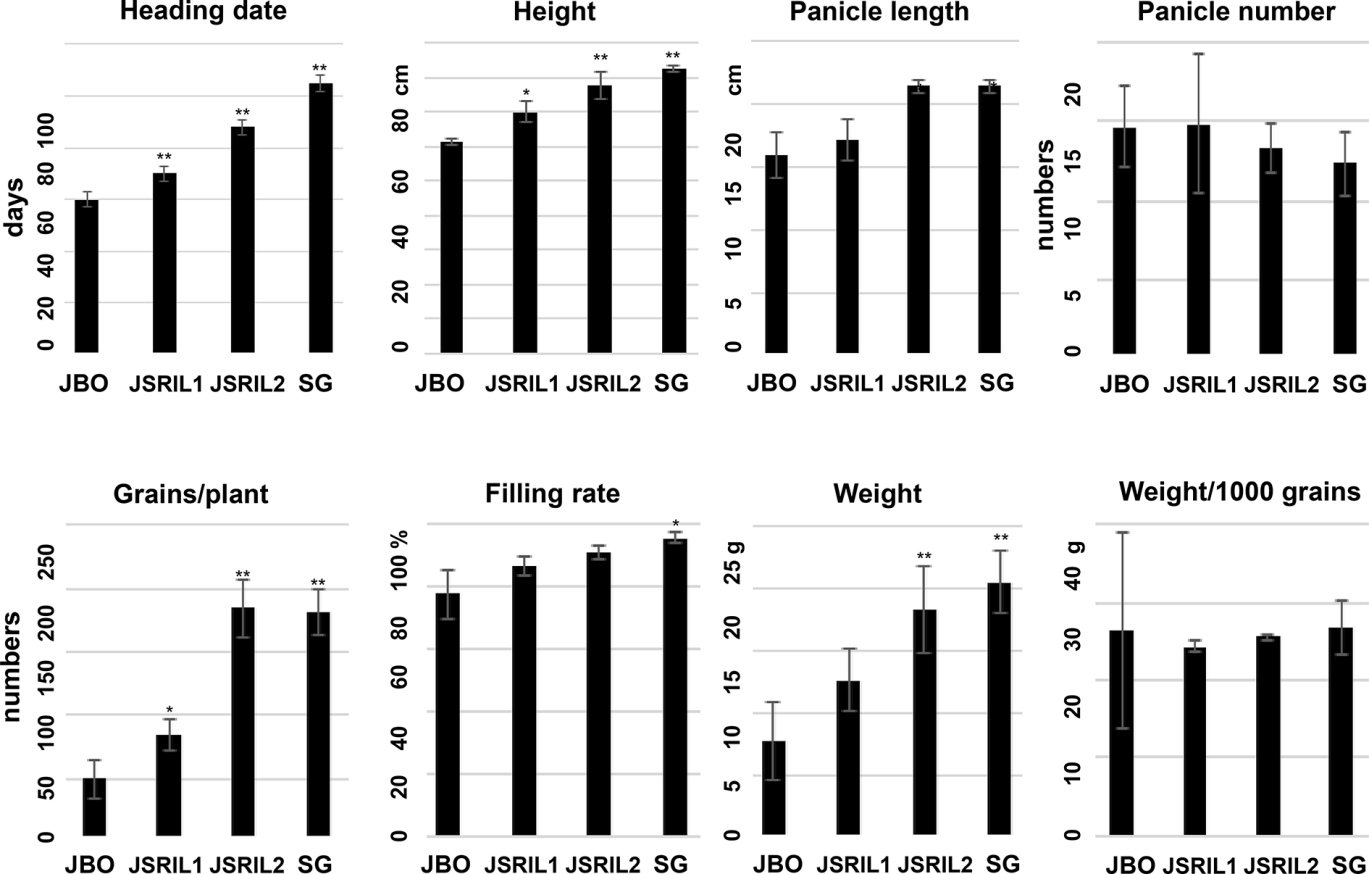
Heading date and yield factors, such as height, panicle length, panicle number, number of grains per plant, filling rate, weight and weight/1000 grains per panicle. Heading dates are given for 15–20 plants, and other factors are given for 3–5 plants of JBO, JSRIL1, JSRIL2, and SG. A one-tailed t test was performed. **p values < 0.01 and *p values < 0.05 indicate significant differences.

To determine how genetic variation affects heading time in cultivated rice, JBO and SG were selected as the female and male parental lines, respectively. JBO has a heading date of Jul. 4 or 57 days after sowing (DAS) in Jeonju, Korea, whereas SG has a heading date of Aug. 17 or 101 DAS under natural field conditions (Fig. S1). Approximately 400 RILs were generated from these parents. The RILs have currently produced more than 8 generations, and traits related to panicle development and other yield factors have been documented (Fig. 1). The RILs have various heading times, ranging from Jul. 1 to Aug. 25. We selected 2 representative RILs: the characteristics of JSRIL1 are more similar to those of JBO, including the heading date, and it flowers around Jul. 14 or 67 DAS, whereas the characteristics of JSRIL2 are more similar to those of SG, and it flowers around Aug. 1 or 85 DAS. A one-tailed t test revealed that all the p values were less than 0.01, suggesting that the differences in heading days were significant. JBO, JSRIL1, and JSRIL2 flowered 44, 34 and 16 days earlier, respectively, than did the SG line under natural paddy field conditions (Fig. S1).

Other yield factors, such as the height, panicle length, panicle number, grain number/plant, filling rate, total weight, and weight/1000 grains, were also measured (Fig. 1). Except for the panicle number and weight/1000 grains, the other yield factors are proportional to the heading date, suggesting that heading date could be a key factor among parental types and JSRILs. This finding confirms that heading days are important for guaranteeing sufficient vegetative growth and a proper filling period (40–45 days) after heading in paddy fields as winter approaches (Izawa 2007; Cho et al. 2017).

### SNPs are unequally distributed among chromosomes in the parental lines and RILs

Genomic DNA was prepared as described in the Methods section and sequenced on the Illumina HiSeq 4000 platform (Illumina.com) via a proprietary reversible terminator-based method. Approximately 14 Gb (40× genome size) of sequencing data were obtained from the wild-type parents and 2 JSRILs (Table S1). The reference genome assembly (IRGSP-1.0_genome, RefN) of rice, *O. sativa* spp. japonica cv. Nipponbare, was downloaded from the Rice Annotation Project (RAP) database (http://rapdb.lab.nig.ac.jp). To add to the SNP analysis, the sequences of two representative cultivars with short life cycles, *O. sativa* spp. japonica cv. Kitaake, Aus cv. Kasalath, and Indica cv. HR-12, were downloaded from the NCBI SRA (https://www.ncbi.nlm.nih.gov/sra/). In addition, another Nipponbare sequence (Nipponbare1, SRR1043564) was downloaded to determine the number of spontaneous SNPs.

Sequencing reads were mapped to RefN, and variant calling was performed and deposited at a shared database (https://figshare.com/). The local realignment of reads around insertions/deletions (indels) was analyzed as described in the Methods. Sequence variations and their effects were analyzed with SnpEff 4.3i (https://pcingola.github.io/SnpEff/). In total, 19,930 insertions (Ins) and 21,874 deletions (Dels) were detected. The number of 0/1 SNPs in the studied lines ranged from 10,000–26,000, with JSRIL2 having the highest number of SNPs at 25,954. The numbers of 1/1 SNPs in parental types JBO and SG were 29,994 and 27,314, respectively, whereas those in JSRIL1 and 2 were 23,605 and 21,255, respectively, suggesting that the parental types have unique SNPs that differed during breeding processes. In total, 667,607 SNPs were detected (Table S2).

**Fig. 2:**
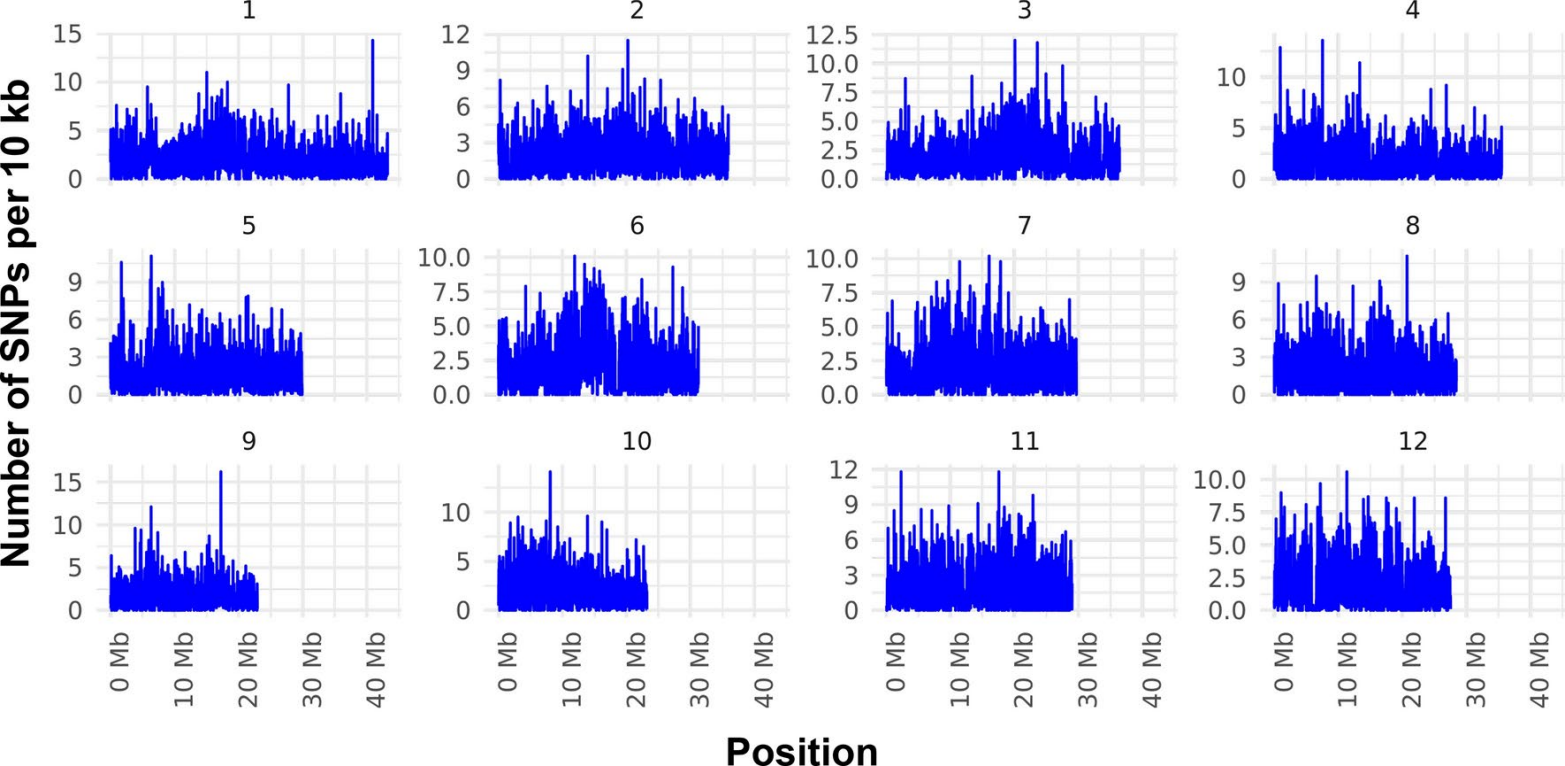
Number of SNPs per 10 kb on chromosomes. SNPs are unequally distributed along chromosomes among the parental lines and RILs. In total, 667,607 SNPs were detected differently among the chromosomes. Chr1 had the most SNPs, with 79,522, whereas Chr9 contained 38,367 SNPs. In addition, Chr4 presented 1 SNP per 649 bp, whereas Chr10 presented 1 SNP per 492 bp

Overall, the SNPs covered the entire chromosome, with an average of one SNP per 560 bp (Fig. 2 and Table S3). However, the number of SNPs varied among the chromosomes. Chr1 had the most SNPs, with 79,522, whereas Chr9 contained the fewest SNPs, with 38,367. In terms of the SNP rate, Chr4 presented 1 SNP per 649 bp, whereas Chr10 presented 1 SNP per 492 bp (Table S3). SNPs were classified according to whether they occurred in genic or intergenic regions, and the number of SNPs was analyzed via sequence ontology (SO) to assess sequence changes and impacts. Most variations were found in intergenic (47.8%) and upstream (14.9%) regions, whereas fewer (1.9%) were found in downstream regions. In genic regions, SNPs and indels were found in exons (6.8%) and introns, including splice sites (8.3%). However, the number of SNPs per 10 kb was highest in exons, at 79.0 SNPs/10 kb, followed by the downstream 300-bp region, at 47.3 SNPs/10 kb (data not shown). Introns and the upstream 2-kb regions presented similar rates, ranging from approximately 31.1 to 36.5 SNPs/10 kb. The rate in genic regions was much higher than that in intergenic regions, at 11.5 SNPs/10 kb. These data suggest that the SNPs in genic regions are exploited more often during crossing based on phenotypic observations.

### Parental genotypes contributed unevenly to JSRILs across chromosomes

**Fig. 3:**
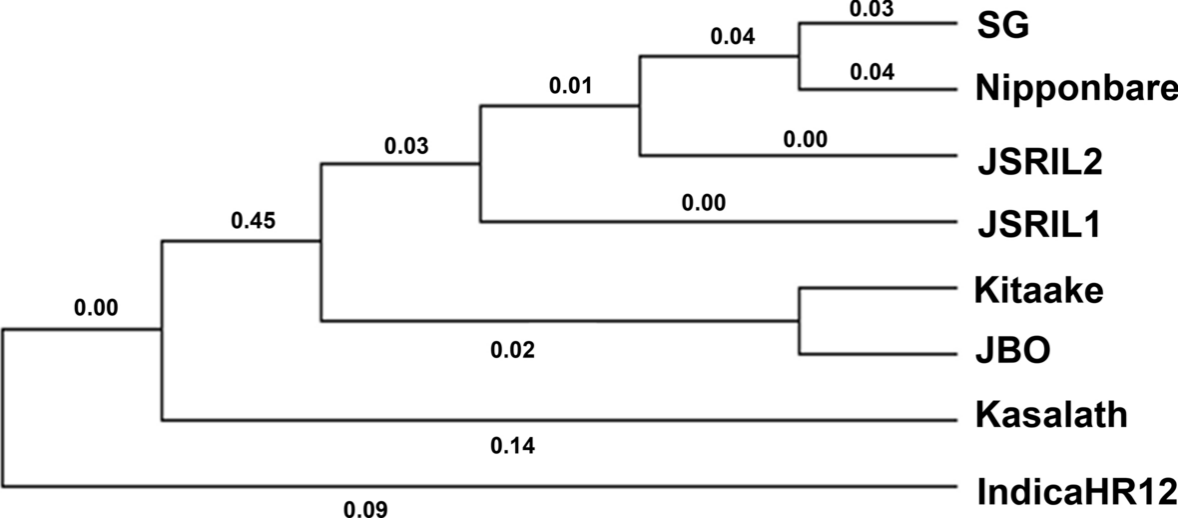
A phylogenetic tree of JBO, SG, their RILs, Nipponbare, Kasalath, Kitaake, and IndicaHR12 was generated with SNPhylo (Lee et al. 2014). A vcf file containing 667,607 SNPs was used. These findings indicate that the SG is closer to Nipponbare1 than the RILs are. Compared with the others, JBO is phylogenetically farther, suggesting that its genetic background is quite unique. Correlation information was generated in Newick format from SNPhylo and transformed into a network, and circular tree diagrams were generated via the Molecular Evolutionary Genetics Analysis program with the toggle scale option (MEGA XI; Tamura et al. 2021)

To determine the evolutionary relationships, a phylogenetic tree was generated with SNPhylo (Fig. 3). The phylogenetic tree revealed that SG was located closer to Nipponbare than to the JSRILs. JBO was more distant from the other lines and was part of the same clade as Kitaake was, suggesting that its genetic background is unique. Kasalath and Indica were outgrouped as expected.

**Fig. 4:**
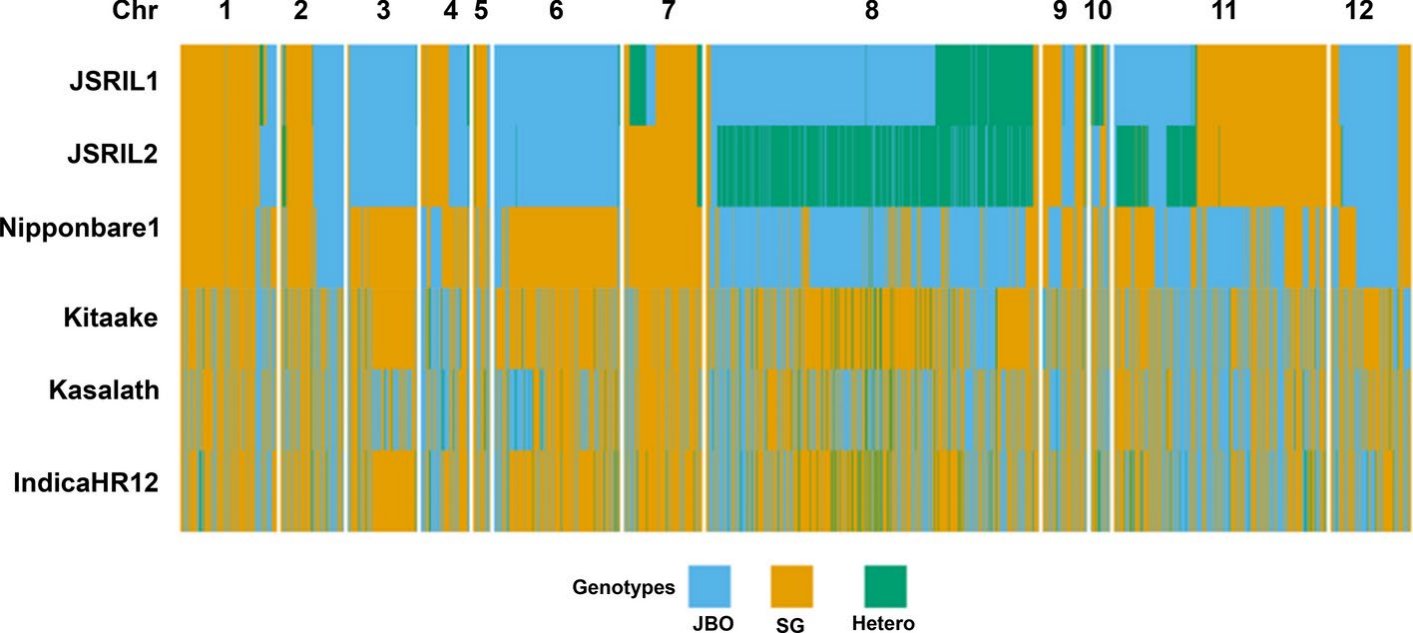
Graphical representations of ABH genotypes of RILs. Genotypes are shown in blue (JBO), orange (SG), and green (heterozygous) depending on the parental genotype. The parental contribution for each chromosome ranged from 42–46% among the JSRILs. However, the contribution rates of the parents to the chromosomes (Chrs) among the JSRILs widely varied. Almost equal contributions were found for Chrs 2, 4, 10, and 11 of JSRIL1. In contrast, the maternal JB contributions were greater for Chrs 3, 7, 8 and 12, whereas the paternal SG contributions were greater for Chrs 1, 5, 7, 9, and 11. Among the heterozygous genotypes, Chr8 and Chr11 presented higher rates of heterozygosity in JSRIL2 than in JSRIL1

To delineate how the genetic components of the parental types, JBO and SG, contributed to those of the JSRILs, the SNP genotypes were converted to ABH genotypes for JBO (Parent A, A), SG (Parent B, B), and the heterozygous genotype (H). The parental contribution for each chromosome varied between the JSRILs. The JSRIL1 genomic makeup was 46.4% from JBO and 43.1% from SG, making maternal JBO the main contributor, whereas the JSRIL2 genomic makeup was 42.4% from JBO and 46.6% from SG (Table 1). These results reflect the physical and heading date characteristics of the JSRILs compared with those of the parental types (Fig. 1). Interestingly, the contribution rates of the parents to the individual chromosomes (Chrs) in the JSRILs widely varied (Table 1 and Fig. 4). In JSRIL1, largely equal parental contributions were found in Chrs 2, 4, 10, and 11; in contrast, Chrs 1, 3, 5, 6, 7, 8, 9, 11, and 12 had preferential contributions from one of the parental types. Specifically, Chrs 3, 7, 8 and 12 are primarily from the maternal JBO line, whereas Chrs 1, 5, 7, 9, and 11 are primarily from the paternal SG line. Interestingly, very similar preferential chromosome contributions were found in JSRIL2, although Chr7 contained a much greater contribution from SG (92.8%) than that in JSRIL1 (60.5%). In terms of the heterozygous genotypes, Chr8 and Chr11 had higher rates of heterozygosity in JSRIL2 than in JSRIL1. These results suggest that chromosome recombination occurs such that parental genotypes contribute unevenly across chromosomes, resulting in phenotypic differences between the RILs. In addition, chromosomal mixing occurs, but shuffling occurs on a larger scale of up to several dozen megabases, as observed in many genetic studies. These chromosomal biases may also occur because RILs are selected based on heading date.

**Table 1:** Parental contributions to JSRIL1 and JSRIL2

	JSRIL1			JSRIL2		
Chr	JBO (%)	SG (%)	Hetero (%)	JBO (%)	SG (%)	Hetero (%)
Chr01	11.4	85.2	3.3	18.0	81.9	0.1
Chr02	51.7	46.1	2.2	49.1	45.6	5.3
Chr03	95.6	2.8	1.7	96.3	2.8	0.9
Chr04	38.2	57.0	4.8	41.6	57.0	1.4
Chr05	17.2	82.7	0.1	17.2	81.2	1.6
Chr06	97.8	0.0	2.2	99.4	0.0	0.6
Chr07	13.6	60.5	25.9	0.3	92.8	6.9
Chr08	67.8	3.1	29.1	12.5	3.1	84.4
Chr09	31.5	66.9	1.6	31.5	66.7	1.8
Chr10	21.5	24.9	53.6	63.6	36.0	0.4
Chr11	37.8	61.3	0.9	10.2	61.9	27.9
Chr12	73.0	26.9	0.1	68.8	30.0	1.2
Average	46.4	43.1	10.5	42.4	46.6	11.0

The chromosomal contributions suggest that Nipponbare, Kitaake, Kasalath and Indica share contributions with JBO and SG, suggesting that these lines share an origin and were selected during breeding processes (Fig. 4). The phylogenetic analysis was expanded to include KRICE_CORE (Kim et al. 2016) generated from whole-genome resequencing of the 137 rice lines in the mini core collection (Fig. S2). These lines include domestically adapted weedy and landrace rice and bred lines, as well as lines from Africa, Europe, and America. JBO and SG belong to clades with Jejubukjeju-2002–340 and Gou 405 in the KRICE_CORE collection, respectively. SG is closely positioned to Nipponbare1 and RefN (IRGSP-1.0_genome)**,** suggesting that it is not very different from varieties such as Gou 405, Iri336 and Suwon 301. Kasalath and Kitaake belong to the clades of BELLARDONE and PUKHI, respectively, and are more closely related to the clade that includes Indica HR12. The distances in clade positions of JBO, JSRIL1 and JSRIL2 from SG suggest that multiple recombination and introgression events in the chromosomal regions occurred among the rice cultivar groups and that parental JBO and SG genes were generated from those of their original ancestors.

### Genic regions of heading-related genes in RILs are delineated by SNPs

Heading is one of the main agronomic traits associated with many yield factors, as shown in Fig. 1. We examined how parental alleles contribute to RIL heading. Seventy genes have been reported to be involved in determining heading day in rice (Cho et al. 2017; Zhou et al. 2021). The SNPs in the parental lines and JSRILs were searched along with those of the Kasalath, Kitaake and Indica cultivars (Table S4 and Fig. S3). The variation in SNPs around many known flower-inducing genes was minimal. Among the 71 genes reported by Zhou et al. (2021), 63 genes, such as *OsGI, Ghd7, Ehd1, Days to heading on chromosome 2* (*DTH2), DTH3, Ehd2, Ehd3,* and *RFT1*, presented no SNPs in JBO, SG, or the JSRILs.

In JSRIL1 and JSRIL2, 5 genes, *Hd1, Hd3a, SDG711, OsVIL2*, and *OsCRY1b*, were inherited from the female parent JBO. Compared with other representative early-flowering cultivars (Table S4), *Hd1* and *SDG711* presented the highest similarity to Kasalath, whereas *OsVIL2, OsRR1*, and *OsCRY1b* presented the highest similarity to Kitaake; *Hd3a* originated from an unidentified source that is likely to be Nipponbare. In contrast, *OsFKF1* and *OsSET33* were derived from the male parent SG, and these genes were similar to those of Nipponbare, Kasalath, or Kitaake. Interestingly, JSRIL1 carried the paternal *OsHESO1* gene, whereas JSRIL2 carried a maternal copy of this gene.

As shown below, allele combinations appeared to be associated with the heading date of JSRILs. Thus, the early heading of JSRIL1 and JSRIL2 might be due to the effects of the photoperiodic genes *Hd1, Hd3a, SDG711, OsVIL2,* and *OsCRY1b* from the maternal JBO line and heading may be delayed by *OsFKF1* and *OsSET33* from the paternal SG line (Fig. S3 and Table S4).

### Gene expression profiling by RNA-seq

To evaluate genome-wide gene expression in floral transition stages, total RNA was extracted from the JBO, JSRIL1, JSRIL2 and SG lines at approximately 10 a.m. on June 23 (46 DAS), July 6 (59 DAS), July 13 (66 DAS), and July 20 (73 DAS), respectively, approximately 20–40 days before heading. Given that many photoperiodic genes are diurnal, samples were also collected at midnight on June 22 (45 DASN). In a previous report, the expression of florigens such as *Hd3a* and *RFT1* in LD flowering lines such as Norin 8 and Dongjin reportedly increased 30–35 days (70 DAS) before heading, whereas the expression of *MADS-box transcription factor 14* (*OsMADS14)* and *OsMADS15*, which are downstream of *Hd3a* and *RFT1*, increased in the inflorescence meristem starting in the primary panicle branch generation stage (Komiya et al. 2008, 2009). RNA from the SG line collected on May 22 (20 DAS) was extracted as a reference for RNA-seq. RNA-seq was performed as described in the Methods section. All the transcript values were increased by 1 to avoid NA error in the program (Table S5). According to the RAP database (http://rapdb.lab.nig.ac.jp), 53,075 transcripts, including 8056 predicted transcripts, were annotated in the genome. More than 31,347 to 34,855 ts were counted at least once, and their median counts ranged from 59.7 to 77.3. Interestingly, the number of counts from midnight (45 DASN) ranged from 109.8 to 114.2 and was higher than the number of daytime counts by approximately 40, suggesting that the expression of many genes was modulated at night. Compared with SG at 20 DAS, 24,688 ts were modulated according to the criteria of twofold up- or downregulation, with adjusted *p* values less than 0.05 and counts greater than 30 (Fig. S4). Approximately 18,000 to 22,000 ts were not counted at all during the time window.

**Fig. 5:**
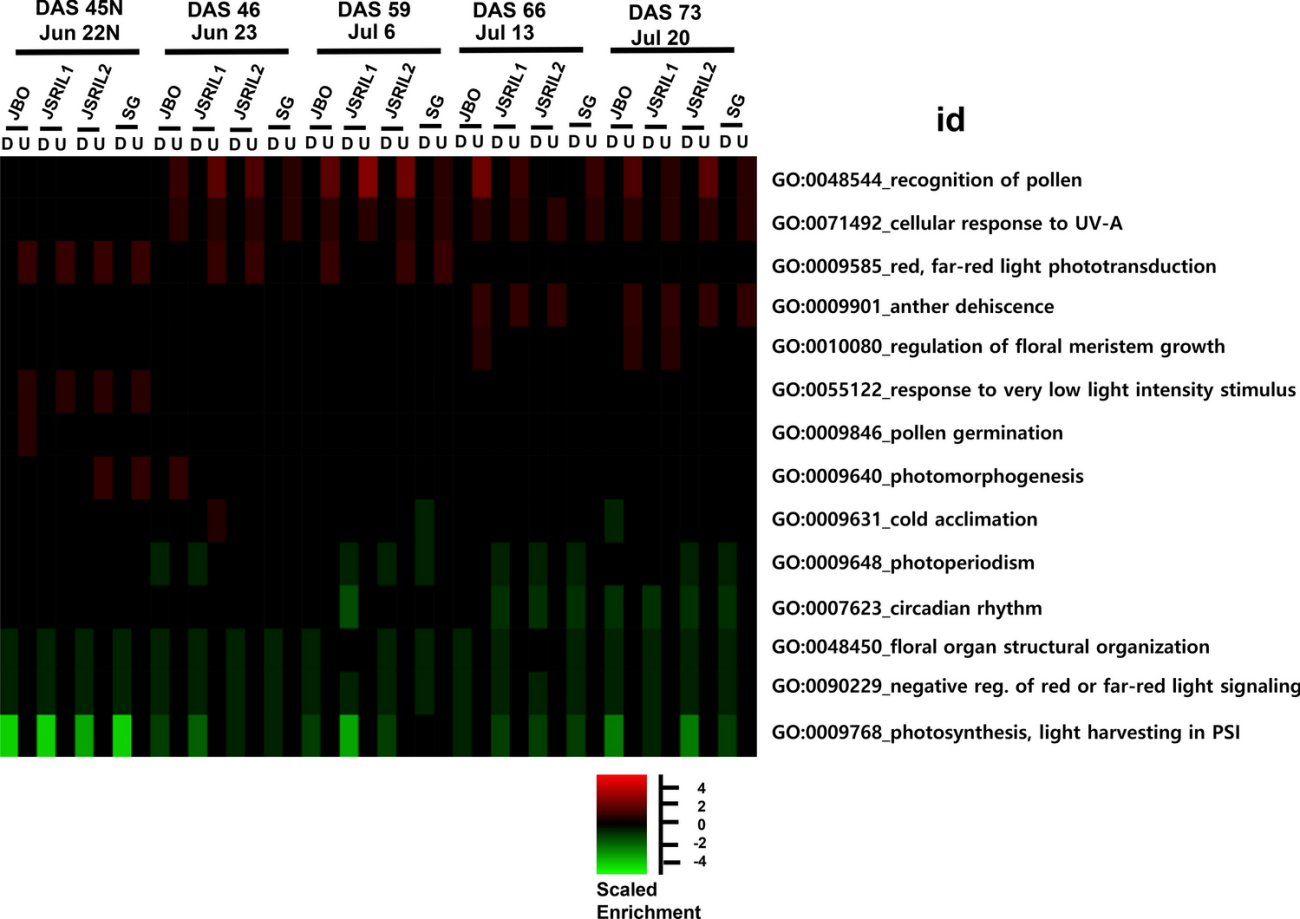
Enriched GO terms. Gene Ontology (GO) enrichment analysis of the genes was performed as described in the Methods section. D: The enriched terms for downregulated genes are underlined, and the genes are color coded in green. U: The enriched terms for upregulated genes are shown, and the genes are color coded in red. The adjusted *P* values for the GO enrichment analysis are scaled from 0 to  − 5 or  − 5 for the up- and downregulated genes, respectively (scaled enrichment)

The enrichment of gene ontology (GO) terms associated with up- or downregulated transcripts was examined as described in the Methods section. At midnight at 45 DAS (45 DASN), the downregulated and upregulated genes were enriched in photosynthesis, light harvesting in photosystem I (GO:0009768) and response to very low light intensity stimulus (GO:0055122), respectively, as expected (Fig. 5 and Table S6). At 46 DAS, the upregulated genes were enriched in red, far-red light phototransduction (GO:0009585), the cellular response to UV-A (GO:0071492) and photomorphogenesis (GO:0009640), whereas the downregulated genes were enriched in floral organ structural organization (GO:0048450). At 59 DAS, the upregulated genes were enriched in recognition of pollen (GO:0048544), detection of visible light (GO:0009584), secondary growth (GO:0080117), and multicellular organism growth (GO:0035264), whereas the downregulated genes were enriched in photoperiodism (GO:0009648) and cold acclimation (GO:0009631). At 66 DAS, the upregulated genes were enriched in the terms “cellular response to high light intensity” (GO:0071486), “cellular response to UV-A” (GO:0071492), “anther dehiscence” (GO:0009901), and “auxin biosynthetic process” (GO:0009851), whereas the downregulated genes were enriched in “pyridine nucleotide catabolic process” (GO:0019364), “ADP catabolic process” (GO:0046032), “photoperiodism” (GO:0009648), “internode patterning” (GO:0080006), and “regulation of root meristem growth” (GO:0010082). At 73 DAS, the upregulated genes were enriched in recognition of pollen (GO:0048544), protein maturation by protein folding (GO:0022417) and regulation of floral meristem growth (GO:0010080), whereas the downregulated genes were enriched in circadian rhythm (GO:0007623), negative regulation of red or far-red light signaling pathway (GO:0090229) and regulation of root meristem growth (GO:0010082). Overall, GO terms such as photoperiodism, cold acclimation, floral organ structural organization, and anther dehiscence were enriched, suggesting that many genes related to floral development are modulated by up- or downregulation compared with vegetative growth in the late-flowering SG at 20 DAS.

### The expression of florigens such as *Hd3and RFT1*, and *MADS14* is roughly proportional to the heading days of parental lines and RILs

**Fig. 6:**
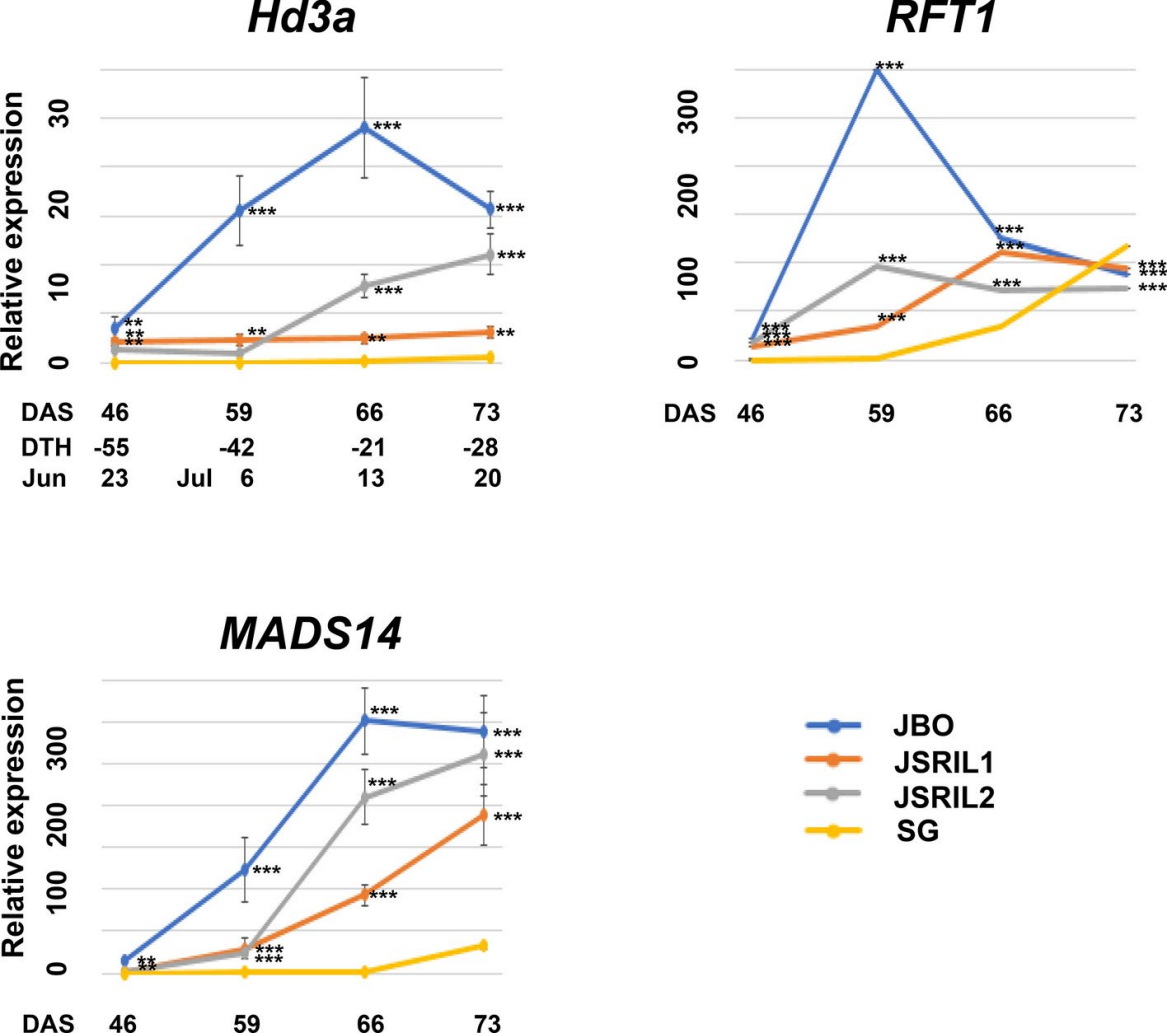
qRT‒PCR analysis of florigens and *OsMADS14* at 46, 59, 66 and 73 DAS. JBO is a japonica rice cultivar with a heading date of Jul. 4 or 57 days after sowing (DAS) in Jeonju, Korea (35.83 N 127.05 E). JSRIL1 has more characteristics similar to those of JBO in terms of the heading date and flowers around Jul. 14, or 66 DAS, and JSRIL2 flowers around Aug. 1 or 85 DAS. SG is a japonica rice cultivar whose heading date is Aug. 17 or 101 DAS under natural field conditions (Fig. S1). These days are also referenced at 101 days of the SG as days to heading (DTH) and dates. Fifteen to twenty plants were observed for heading in the paddy fields. Dunnett’s test was performed to determine the significance of the differences, with the result for SG at the same DAS used as a control. The Dunnett’s test *p* values are as follows: 0; ‘***’, 0.001; ‘**’, 0.01; and ‘*’, 0.05

The expression patterns of *Hd3a* in JBO, JSRIL1, and JSRIL2 on each day were compared with those in SG at 20 DAS, as described in the Methods section (Fig. 6). SG is a middle- to late-maturation rice cultivar that experiences heading at approximately 101 DAS. At 46 DAS, the expression of *Hd3a* increased 3.4-, 2.1-, and 1.4-fold in JBO, JSRIL1, and JSRIL2, respectively, compared with that in SG, suggesting that florigen expression was triggered in JBO and JSRIL1 at that time. This difference was significant according to Dunnett’s test, as described in the Methods section. JBO showed the greatest increase of 24-fold at 66 DAS, followed by a 15.6-fold increase at 73 DAS. In JSRIL1, *Hd3a* expression was maintained at approximately 2.1-fold higher levels than that in SG up to 66 DAS, reached 3.1-fold higher levels at 73 DAS, and then stabilized. In JSRIL2, heading occurred 18 days later than that in JSRIL1, and *Hd3a* levels were maintained until 59 DAS; however, *Hd3a* increased 7.8- and 11.1-fold at 66 and 73 DAS, respectively. These values are much greater than those of JSRIL1, suggesting that other factors might be involved in flower induction. However, *Hd3a* expression in SG was undetectable until 73 DAS and remained weak. These data suggest that *Hd3a* is a major floral activator that plays a central role in regulating heading time under NLC and is the main florigen under SD conditions (Komiya et al. 2008).

*RFT1*, the closest homolog to *Hd3a*, increased 21.66-, 13.21-, 18.04-, and 0.20-fold in JBO, JSRIL1, JSRIL2, and SG, respectively, at 46 DAS, suggesting that floral meristems are initiated in JBO and RILs (Fig. 6); these expression levels increased 298.9-, 34.9-, and 97.0-fold at 59 DAS and 126.2-, 111.2- and 72.0-fold at 66 DAS, respectively. The expression was maximal at 66 DAS and 59 DAS for JSRIL1 and JSRIL2, respectively. In SG, *RFT1* expression increased 34.4-fold at 66 DAS, suggesting that floral transition had begun. During this period, the induction of *RFT1* was tenfold greater than that of *Hd3a*, suggesting that *RFT1* might be a main florigen under NLC, as previously reported for LD conditions (Komiya et al. 2009).

Hd3a interacts with a 14-3-3 protein in the cytoplasm of the apical cells of shoots, translocates to the nucleus and binds the bZIP transcription factor FD to form the florigen activation complex (FAC) (Tsuji et al. 2013). This complex regulates the expression of the *APETALA1 (AP1)/FRUITFULL (FUL)-*like genes *MADS14, MADS15*, and *MADS18. MADS14* appears to coordinate to specify the identity of an inflorescence meristem downstream of the florigen signal. *MADS14* expression increased 13.94-, 0.41-, 1.61-, and 0.1-fold at 46 DAS in JBO, JSRIL1, JSRIL2, and SG, respectively, suggesting that the floral meristem in JBO was already initiated (Fig. 6). The expression levels were 122.58-, 29.17-, 23.05-, and 0.18-fold greater at 59 DAS in JBO, JSRIL1, JSRIL2, and SG, respectively, suggesting that floral meristems were initiated in JSRILs. *MADS14* expression in JBO reached the greatest increase of 301-fold and 289-fold at 66 DAS and 73 DAS, respectively. The expression of *MADS14* in JSRIL2, JSRIL1, and SG increased continuously until 73 DAS (July 20), increasing by 261-fold, 188-fold, and 32-fold, respectively. Because *Hd3a* and *RFT1* expression was increased in JBO, JSRIL1 and JSRIL2 but *RFT1* expression was increased in SG, these plants were under LD conditions. This induction time frame and expression level explain the parental types and JSRILs.

### Gene expression of photoperiodic genes with no SNPs among JSRILs

As described above, SNP analysis of photoperiodic genes in the RILs (Fig. S3 and Table S4) revealed that the genes containing no SNPs (63 genes), such as *OsGI, Ghd7* and *Ehd1,* were not different among the lines, but the expression levels were consistent with heading days in the neutral, suppression and activation modes. The genes in the maternal JBO group (5 genes), namely, *Hd1, Hd3a, OsVIL2, SDG711*, and *OsCRY1b*, might be involved in accelerating heading, whereas the genes in the paternal SG group (2 genes), namely, *SDG723* and *OsFKF1,* might be involved in delayed heading among RILs. The biased gene, *OsHESO1,* is reversed, with JSRIL1 and JSRIL2 containing the paternal and maternal genes for the heading day, respectively.

**Fig. 7:**
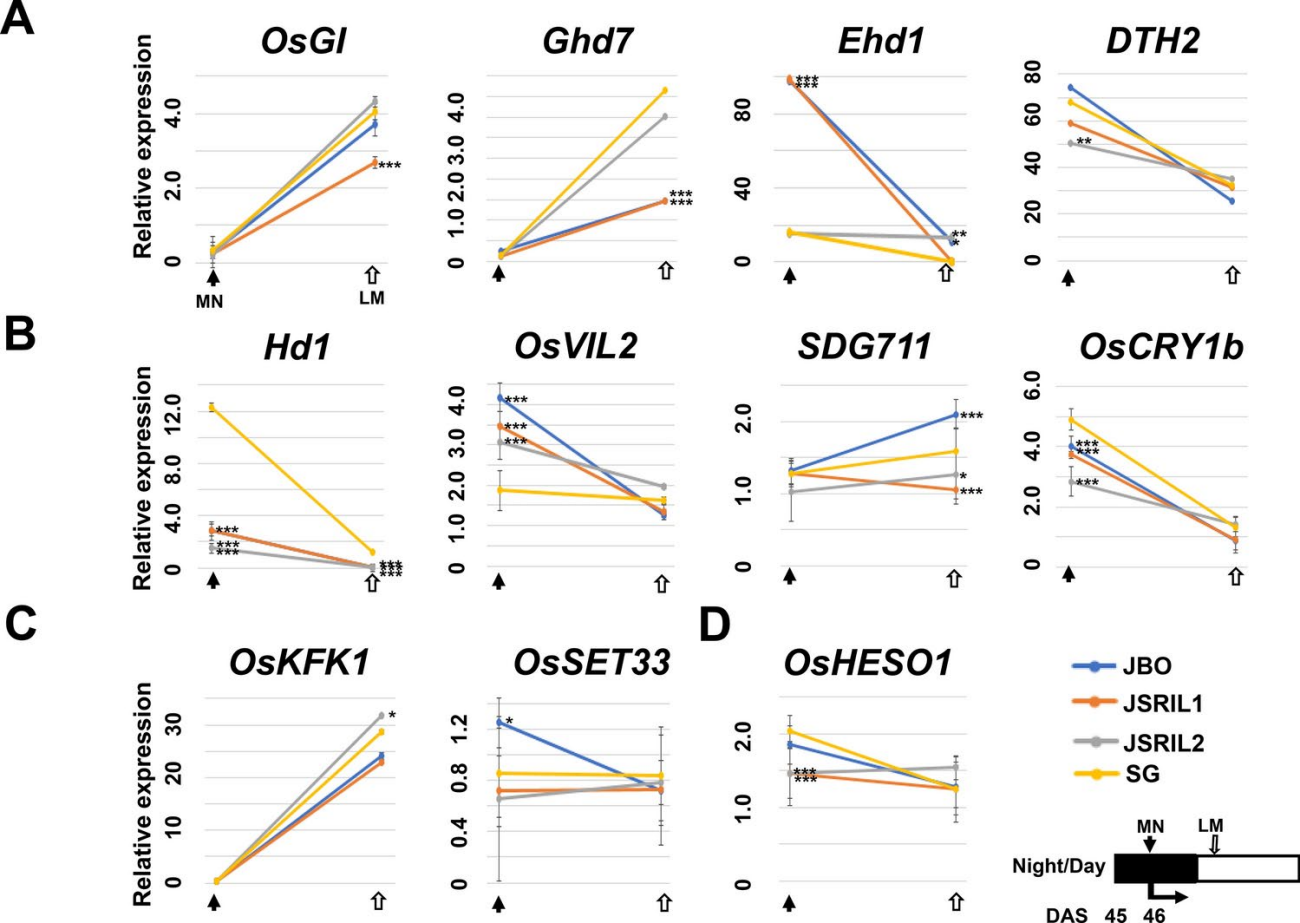
qRT‒PCR analysis of JSRIL and parental genotypes at midnight at 45 DAS (MN) and late morning at 46 DAS (LM). Given that the gene expression of many photoperiodicity genes is diurnal, total RNA was prepared at the indicated times. Among the 71 genes reported by Zhou et al. 63 genes had no SNPs among the parental types and JSRILs. Only 4 genes were tested: *OsGI, Ghd7, Ehd1,* and *DTH2* (**A**); JSRIL1 and JSRIL2 have maternal JBO genes, such as *Hd1, OsVIL2, SDG711*, and *OsCRY1b* (**B**); and *OsFKF1* and *OsSET33* are from the paternal SG line (**C**). The parent-dependent *OsHESO1* gene was from the paternal line in JSRIL1 and the maternal line in JSRIL2 (**D**). These groups were classified on the basis of SNP analysis (Table S4 and Fig. S3). Dunnett’s test was performed to determine the significance of the differences, with the results for SG at the same DAS at MN and LM used as controls. Night and day are scaled according to June 23rd in Jeonju, Korea (35.83 N, 127.05 E). The Dunnett’s test *p* values are as follows: 0; ‘***’, 0.001; ‘**’, 0.01; and ‘*’, 0.05

These data suggest that the induction of *Hd3a, RFT1* and *MADS14* in JBO and RILs has already been initiated at approximately 46 DAS (Fig. 6). We tested the expression of photoperiodic genes at midnight at 45 DAS (MN) and 10 am at 46 DAS (late morning [LM]) in these lines. Among the 63 genes without SNPs, *OsGI, Ghd7, Ehd1,* and *DTH2* were chosen for qRT‒PCR (Fig. 7A). In *Arabidopsis*, GI is a flowering time-related gene that is strongly involved in its circadian clock system (Fowler et al. 1999). In contrast, the rice osgi-1 mutation extended the time to heading only under SD conditions, whereas under LD conditions, osgi-1 mutant plants presented no severe phenotypic changes, but the gene seemed to orchestrate the circadian clock and diurnal rhythms of the global transcriptome, including the genes involved in primary metabolism under natural day–night cycles in rice (Itoh et al. 2010; Izawa et al. 2011). The expression of the gene was marginal at MN in all the lines, as expected. In contrast, under LM, gene expression increased by 3.71-, 2.68-, 4.32-, and 4.06-fold in JBO, JSRIL1, JSRIL2, and SG, respectively, suggesting that the expression of *OsGI* is circadian rhythm-dependent and diurnal.

The increased expression of *Ghd7* under LD conditions delays heading (Xue et al. 2008). *Ghd7*, combined with *Ehd1,* also sets a critical day length for *Hd3a* florigen expression in rice (Itoh et al. 2010). Its expression was marginal in these lines at MN, whereas its expression in LM was 1.5-fold greater in JBO and JSRIL1 (Fig. 7A). In contrast, the expression of *Ghd7* was increased by 3.51- and 4.16-fold in JSRIL2 and SG, respectively. This difference between those of JB and JSRIL1 and those of JSRIL2 and SG in LM was significant according to Dunnett’s test, as described in the Methods section. These data suggest that the inhibitory effects of *Ghd7* on photoperiod signaling are strong and might explain the delayed heading of JSRIL1 and JSRIL2 by 10 and 28 days, respectively, compared with that of JBO.

The *Ehd1* gene promotes heading under LD conditions and may act independently of a functional allele of *Hd1* (Doi et al. 2004). At MN, its expression was increased approximately 98.0-fold in JBO and JSRIL1, whereas it was increased 15.0-fold in JSRIL2 and SG, suggesting that *Ehd1* is involved in early heading (Fig. 7A). Interestingly, in LM, the expression of *Ehd1* in JBO and JSRIL2 was maintained at 10.85 and 13.4, respectively, whereas it was almost completely absent in JSRIL1 and SG. The induction of *Ehd1* in JBO and JSRIL1 and that of *Ghd7* in JSRIL2 and SG clearly indicated that inducer and suppressor expression correlated with the heading date in these lines. However, it is unclear whether these differences are due to genetic differences or temporal differences during these periods.

Recent studies have suggested that *DTH2* might be necessary for the expression of *Hd3a* and *RFT1,* but the *Hd1* and *Ehd1* pathways are independent of florigen genes in rice under LD conditions, suggesting the existence of a more finely tuned photoperiod-sensing mechanism (Wu et al. 2013). *DTH2* was expressed at high levels in the lines: 50.5–74.2-fold higher at MN (Fig. 7A) and 25.5–35.1-fold higher in LM. *DTH3* was increased 2.8–3.3-fold at MN and 0.9–1.9-fold in LM, but its expression was mixed (data not shown). These data suggest that the expression of these genes is diurnal and remains to be elucidated.

### Genes originating from the maternal line in JSRILs are the main contributors to early heading

*Hd1, Hd3a, OsVIL2, SDG711*, and *OsCRY1b* in JSRILs originated from the maternal JBO line (Fig. S3 and Table S4). *Hd1* in JSRIL1 and JSRIL2 originated from JBO and was more similar to that of Kasalath (recessive *hd1*), and its expression was marginal over time. Under LD conditions, functional *Hd1* acts as an inhibitor (Lin et al. 2000; Yano et al. 2000). At MN, *Hd1* expression was increased 12.3-fold, whereas *hd1* expression in JBO and JSRILs was decreased 3–fourfold (Fig. 7B). This finding was significant according to Dunnett’s test. In addition, the expression of this gene in LM throughout the study period was greater (data not shown) than that of other genes, which appeared to cause the late heading of SGs, whereas *hd1* expression in JBO, JSRIL1 and JSRIL2 may explain the early heading in these lines.

The expression of *OsVIL2*, a chromatin remodeling factor, was greater in JBO and the RILs at MN (Fig. 7B). *OsVIL2* expression in JBO and JSRIL was 3.1–4.2-fold greater than that in SG, and this difference was significant according to Dunnett’s test. This increased expression of *OsVIL2* apparently induced heading via the repression of downstream genes. Among the JSRILs, *OsVIL2* was from the maternal JBO line and was the same as that of Nipponbare (Table S4). The paternal SG *OsVIL2* is the same as that of Kasalath. In this study, *OsVIL2* of JSRIL1 and JSRIL2 originated from the maternal JBO line. In an extended comparison, *OsVIL2* was closest to the sequences of Nipponbare and Kitaake, suggesting that these key genes originated from different sources of *hd1*.

In JSRILs, *SDG711*, a key PRC2 subunit that is required for H3K27me3, is derived from the maternal JBO line and is the same as that of Kasalath. Its expression in JBO in LM was twofold greater than that in the control. However, the expression in JSRILs was marginal and could not be induced at 46 DAS. These observations suggest that *SDG711* functions as an activator under NLCs.

The *OsCRY1b* gene is involved in the response to blue light and inhibits coleoptile growth (Hirose et al. 2006). At MN, the expression of *OsCRY1b* ranged from 2.8 to 4.9, with the highest expression in SG, whereas in LM, its expression was similar among the lines, which suggests that *OsCRY1b* functions as a suppressor under NLC.

### Genes originating from the paternal line may be involved in delaying the early heading of JSRILs

At MN, the expression of *OsFKF1* was marginal in all lines, whereas in LM, its expression in JBO and JSRIL1 increased 23.0–24.1-fold, whereas that in JSRIL2 and SG increased 28.7–31.9-fold (Fig. 7C), suggesting that its expression is strongly diurnal and roughly similar to that of *OsGI,* which is consistent with previous observations of the OsFKF1-OsGI interaction (Han et al. 2015). This interaction might regulate the blue-light-mediated activation of *Ehd1* through physical interaction with OsCDF1/OsDOF12.

*OsSET33* and *OsFKF1* in JSRIL2 originated from the paternal SG line (Fig. S3 and Table S4). At MN, the expression of *OsSET33* in JBO was 1.2, whereas that in the other lines ranged from 0.5 to − 0.8; in LM, its expression was at the basal level in all lines (Fig. 7C), suggesting that OsSET33 functions as an activator.

### *OsHESO1* is reversed in terms of heading days in JSRILs

*OsHESO1* has been implicated in photoperiod regulation through posttranscriptional processing of miRNAs and siRNAs (Yano et al. 2016). In our study, JSRIL1 had the paternal SG B type, whereas JSRIL2 had the maternal A type (Fig. S3). At MN and LM, the expression of *OsHESO1* increased approximately 1.5–2.0-fold and was similar in expression among the lines (Fig. 7D). Interestingly, the expression of JSRILs at MN was significantly lower than that in the parental types, and this difference was significant according to Dunnett’s test. However, how this gene is involved in controlling photoperiod-related genes remains unclear, and the gene may work with other factor(s) to induce a consistent delay in heading.

## Discussion

As shown above, the variation in the SNPs in florigens and *MADS14* was minimal. *Hd3a* in JSRIL1 and JSRIL2 was inherited from the JBO line. Interestingly, *Hd3a* in the SG line is the same as that in Kasalath and Kitaake, suggesting that photoperiodic control is determined by the regulation of genes (Takahashi et al. 2009). Taken together, the results indicate that the heading times of JBO and the RILs might have been shortened by the selection of LD-responsive genes such as *Hd3a/RFT1* along with upstream SD and LD regulators such as *hd1* and *OsVIL2* during domestication/breeding. Because *OsFKF1* and *OsSET33* came from the male parent, these results suggest that the alleles in the parents were selected during the breeding process. Many genes upstream of photoperiod-related genes, such as *OsGI, Ghd7* and *Ehd1*, had no SNPs in the JSRILs or their parents. *OsGI, Ghd7,* and *Ehd1* have been shown to be critical components in determining rice photoperiodicity. *OsGI* functions as a circadian clock in the expression of *Ehd1* when blue light coincides with the morning phase (Itoh et al. 2010). *Ghd7* expression was acutely induced when phytochrome signals coincided in a photoperiod-dependent manner and repressed *Ehd1*. Under LD conditions, *Ghd7* expression was gated with a photoinducible phase pattern clearly peaking at subjective dawn. No SNPs were detected in this region in the JSRILs, JBO or SG. However, the induction patterns among these lines were consistent with the heading date. At MN, *OsGI* induction was marginal, whereas *Ehd1* induction was much greater in JBO and JSRIL1 than in JSRIL2 and SG. In contrast, in LM, the induction of *Ghd7* in JSRIL2 and SG was much greater than that in JSRIL1 and JBO. The differences in the induction levels of *Ghd7* and *Ehd1* between the pairs of JBO and JSRIL1 and JSRIL2 and SG also indicate that these paired genes are important in setting photoperiodicity (Itoh et al. 2010). These data also suggest that the regulation of these genes is controlled by upstream genes. *OsFKF1* can also upregulate the expression of the floral activator *Ehd2* and downregulate the expression of the floral repressor *Ghd7* in the regulation of *Ehd1* (Han et al. 2015). The OsFKF1-OsGI interaction might regulate the blue light-mediated activation of Ehd1 through physical interaction with OsCDF1/OsDOF12. The blue light-mediated activation of Ehd1 can upregulate *Ehd1* without affecting *Ehd2* and *Ghd7*. These data suggest that its involvement in *Ehd1* regulation is unique in that it functions without affecting *Ghd7*. OsFKF1 likely acts as an autonomous floral activator because it promotes flowering independent of photoperiod. The paternal origin of the gene in the JSRILs might delay early onset among the genes of the photoperiod network.

Notably, *OsHESO1* is reversed in terms of the heading duration in JSRILs. The findings seem contradictory: the paternal gene is found in JSRIL1, and the maternal gene is found in JSRIL2. This difference might influence the induction of *Hd3a, RFT1* and *MADS14* in terms of expression. The expression levels of these downstream genes in the photoperiod network from 59–73 DAS were greater in JSRIL2 than in JSRIL1, although JSRIL2 presented later heading than JSRIL1 did (Fig. 7). *Hd3a* was detected from 66–73 DAS, whereas *RFT1* was detected at 59 DAS, and *MADS14* was detected from 66–77 DAS. In this context, *Hd3a* induction might be important for inducing the inflorescence meristem, which is why *Hd3a* induction responded sharply at approximately 13.5 h during the day according to a previous report (Itoh et al. 2010). If plants recognize only a single photoperiod, seasonal differences could occur. A simple hypothesis is that this gene might be involved in regulating the transition from SD conditions to LD conditions or vice versa. In addition, reversed parental SNPs in JSRILs might cause photoperiodic response perturbations. As *OsHESO1* is involved in posttranscriptional regulation, this gene might regulate other photoperiod-related genes in combination with target genes (Yano et al. 2016). It remains to be determined how *OsHESO1* is precisely involved in the regulation of photoperiodic genes.

Key genes such as *Hd1, Hd2*, and *Hd3a* were identified through quantitative trait locus (QTL) or backcross analysis from a cross between the rice cultivars ‘Nipponbare’ (japonica) and ‘Kasalath’ (aus) (Yano et al. 2000; Lin et al. 2000; Hori et al. 2016). QTL analysis suggested that *Hd1* might be bifunctional, promoting heading under SD conditions and inhibiting heading under LD conditions. A comparison of the sequences of Nipponbare and Kasalath revealed numerous sequence variations (Yano et al. 2000). A 2-bp deletion in the second putative exon in the Kasalath allele results in a premature stop codon, which might result in a nonfunctional *hd1*. The alleles of JBO and JSRILs are very similar to those of Kasalath and might also be nonfunctional because the coding and intron regions of the gene in JBO are from Kasalath, whereas the promoter regions are from other sources. This gene is also different from that of Kitaake. In contrast, the gene in SG is identical to that in the late-flowering cultivar Nipponbare and might be functional. Fusino et al. (2010) extended the DNA sequences of *Hd1* among 60 landraces of Asian cultivated rice (*Oryza sativa* L.) originating from all regions of Asia, which comprised three cultivar groups, indica, japonica, and aus, as well as accessions of *O. rufipogon* (Fujino et al., 2010). The sequence diversity of *Hd1*, the independent selection of loss-of-function mutations, and multiple introgression events in the chromosomal regions surrounding *Hd1* identified among rice cultivar groups clearly suggest that *Hd1*-driven selection plays an important role in adaptation to local areas (Fujino et al. 2004; Ebana et al. 2011). JBO might also have been chosen under these breeding schemes.

Since the complete rice genome was sequenced, genome-wide sequencing has led to the accumulation of databases of SNPs from various rice landraces and cultivars (IRGSP 2005). Information about SNPs might be helpful for identifying genes with alleles specifically related to flowering. Whole-genome sequencing has also revealed SNPs and indels via comparisons of various rice cultivars and landraces with Nipponbare (Yamamoto et al. 2010; Arai-Kichise et al. 2014; Takano et al. 2014; Kim et al. 2018; Wang et al. 2018). Analyses of various rice accessions identified SNPs and provided phenotypic data. GWAS data are available for agronomic traits, including the heading date, plant height, and grain weight metabolite traits, among others (Zhao et al. 2011, 2015; Yano et al. 2016). The analysis of QTLs has also improved our understanding of agronomic traits. In addition, approximately 20 genes related to heading, such as *Hd1, Hd3a, Ehd1,* and *Ghd7,* were subjected to map-based cloning (Hori et al. 2015). Geological surveys of these genes have shown that alleles of these genes are critical in northern cultivation areas (Izawa 2007). Rice lines with deficient or weak alleles of *Ghd7, DTH8, DTH2, Hd16*, and *OsPRR37* are distributed in northern cultivation areas at high latitudes to control the grain-filling period after fertilization. Breeders have chosen favorable alleles to adapt plants to these regions with maximum productivity.

However, the process might be far more complex. Recently, an analysis of more than 14,000 backcrossed individuals derived from multiple crosses of the japonica rice accession Koshihikari (a common parental line) with diverse rice accessions, such as indica, aus, and japonica, revealed that more than 250 QTLs were widely distributed across the rice genome (Hori et al. 2015). More than 100 QTLs corresponding to the genomic positions of heading date genes have been identified in previous studies. In addition, similar numbers of QTLs were detected in different chromosomal regions, which might provide more opportunities to explain the natural variation. Although we focused on known genes, other genes may also be involved in the regulation of photoperiodicity. Weak QTLs might be difficult to find. Our analysis based on SNPs also suggested that the gene expression level might be critical in this regulation. Expression pattern analysis in conjunction with SNP analysis of elite RILs could be helpful in understanding how rice modulates genes according to photoperiodicity.

## Supplementary Information

Below is the link to the electronic supplementary material.

Supplementary file1 (XLSX 22779 kb)

Supplementary file2: **Fig. S1** Heading days of JBO, JSRIL1, JSRIL2, and SG. JBO is a japonica rice cultivar with a heading date of Jul. 4 or 57 days after sowing (DAS) in Jeonju, Korea (35.83 N 127.05 E), whereas SG is a japonica rice cultivar with a heading date of Aug. 17 or 101 DAS under natural field conditions. JSRIL1 flowers around Jul. 14, or 67 DAS, whereas JSRIL2 flowers around Aug. 1 or 85 DAS. Thus, JBO, JSRIL1, and JSRIL2 flowered 44, 34 and 16 days earlier than the SG line did, respectively, under natural conditions. **Fig. S2** Phylogenetic tree construction for JBO, SG and the JSRILs in conjunction with Nipponbare, Kitaake, Kasalath and Indica. The phylogenetic analysis was extended with the Korean rice core set (KRICE_CORE) generated from whole-genome resequencing of the 137 varieties of rice collection or the Korean rice core set (KRICE_CORE, Kim et al. 2016). The database includes domestically adapted weedy and landrace rice and bred lines, as well as introduced lines from Africa, Europe, and America. The abbreviations of the varieties were used for the analysis. RefN: Reference (IRGSP1.0), Nipponbare1: SRR1043564, IndicaHR12: SRR3056468, Kitaake: SRR7789808, and Kasalath: were downloaded from the NCBI SRA database. Seven genomes are noted by arrows. **Fig. S3** Graphical presentation of heading-related genes on chromosomes of JSRIL1 and JSRIL2. Within JSRIL1 and JSRIL2, 5 genes, Hd1, Hd3a, SDG711, OsVIL2, and OsCRY1b, are from the female parent JBO. In contrast, OsFKF1 and OsSET33 were derived from the male parent SG. The OsHESO1 gene of JSRIL1 had a paternal SG copy, whereas JSRIL2 had a maternal JB copy. **Fig. S4** RNA-seq analysis. Total RNA was extracted from a tiller above ground from the JBO, JSRIL1, JSRIL2 and SG lines. The samples were collected at approximately 10 a.m. on June 23 (46 DAS), July 6 (59 DAS), July 13 (66 DAS), and July 20 (73 DAS), approximately 20–40 days before the heading of each line. The SG line on May 22 (20 DAS) was also prepared as a reference. Given that many photoperiodic genes are diurnal, samples were also collected at midnight on June 22 (45 DASN). The experiments were performed in duplicate. RNA-seq analysis was performed as described in the Methods section. In total, 24,688 transcripts were modulated, with absolute values greater than the thresholds of 1.0 for the log2-fold change and 0.05 for the adjusted p value. The gene expression patterns were clustered with the dist and hclust functions and color coded with the heatmap function in the R and Bioconductor programs (https://www.r-project.org/; https://www.bioconductor.org/). The green and red colors represent downregulated and upregulated genes, respectively. (PPTX 4360 kb)

## Data Availability

The genome and RNA-seq data are available at the NCBI SRA under the BioProject: PRJNA1152297.

## Abbreviations

RILs: Recombinant isogenic lines
DAS: Days after sowing
NLC: Natural long-day conditions
