# Supplementary material for: A combination of upstream alleles involved in rice heading hastens natural long-day responses
Source: Genes Genomics. 2024 Nov 20;47(2):245–61. doi: 10.1007/s13258-024-01597-5 (PMC11757646; doi:10.1007/s13258-024-01597-5)
Supplement: Supplementary file 2 — Supplementary file2: Fig. S1 Heading days of JBO, JSRIL1, JSRIL2, and SG. JBO is a japonica rice cultivar with a heading date of Jul. 4 or 57 days after sowing (DAS) in Jeonju, Korea (35.83 N 127.05 E), whereas SG is a japonica rice cultivar with a heading date of Aug. 17 or 101 DAS under natural field conditions. JSRIL1 flowers around Jul. 14, or 67 DAS, whereas JSRIL2 flowers around Aug. 1 or 85 DAS. Thus, JBO, JSRIL1, and JSRIL2 flowered 44, 34 and 16 days earlier than the SG line did, respectively, under natural conditions. Fig. S2 Phylogenetic tree construction for JBO, SG and the JSRILs in conjunction with Nipponbare, Kitaake, Kasalath and Indica. The phylogenetic analysis was extended with the Korean rice core set (KRICE_CORE) generated from whole-genome resequencing of the 137 varieties of rice collection or the Korean rice core set (KRICE_CORE, Kim et al. 2016). The database includes domestically adapted weedy and landrace rice and bred lines, as well as introduced lines from Africa, Europe, and America. The abbreviations of the varieties were used for the analysis. RefN: Reference (IRGSP1.0), Nipponbare1: SRR1043564, IndicaHR12: SRR3056468, Kitaake: SRR7789808, and Kasalath: were downloaded from the NCBI SRA database. Seven genomes are noted by arrows. Fig. S3 Graphical presentation of heading-related genes on chromosomes of JSRIL1 and JSRIL2. Within JSRIL1 and JSRIL2, 5 genes, Hd1, Hd3a, SDG711, OsVIL2, and OsCRY1b, are from the female parent JBO. In contrast, OsFKF1 and OsSET33 were derived from the male parent SG. The OsHESO1 gene of JSRIL1 had a paternal SG copy, whereas JSRIL2 had a maternal JB copy. Fig. S4 RNA-seq analysis. Total RNA was extracted from a tiller above ground from the JBO, JSRIL1, JSRIL2 and SG lines. The samples were collected at approximately 10 a.m. on June 23 (46 DAS), July 6 (59 DAS), July 13 (66 DAS), and July 20 (73 DAS), approximately 20–40 days before the heading of each line. The SG line on May 22 (20 DAS) was also prepared [file 13258_2024_1597_MOESM2_ESM.pptx]

## Slide 1
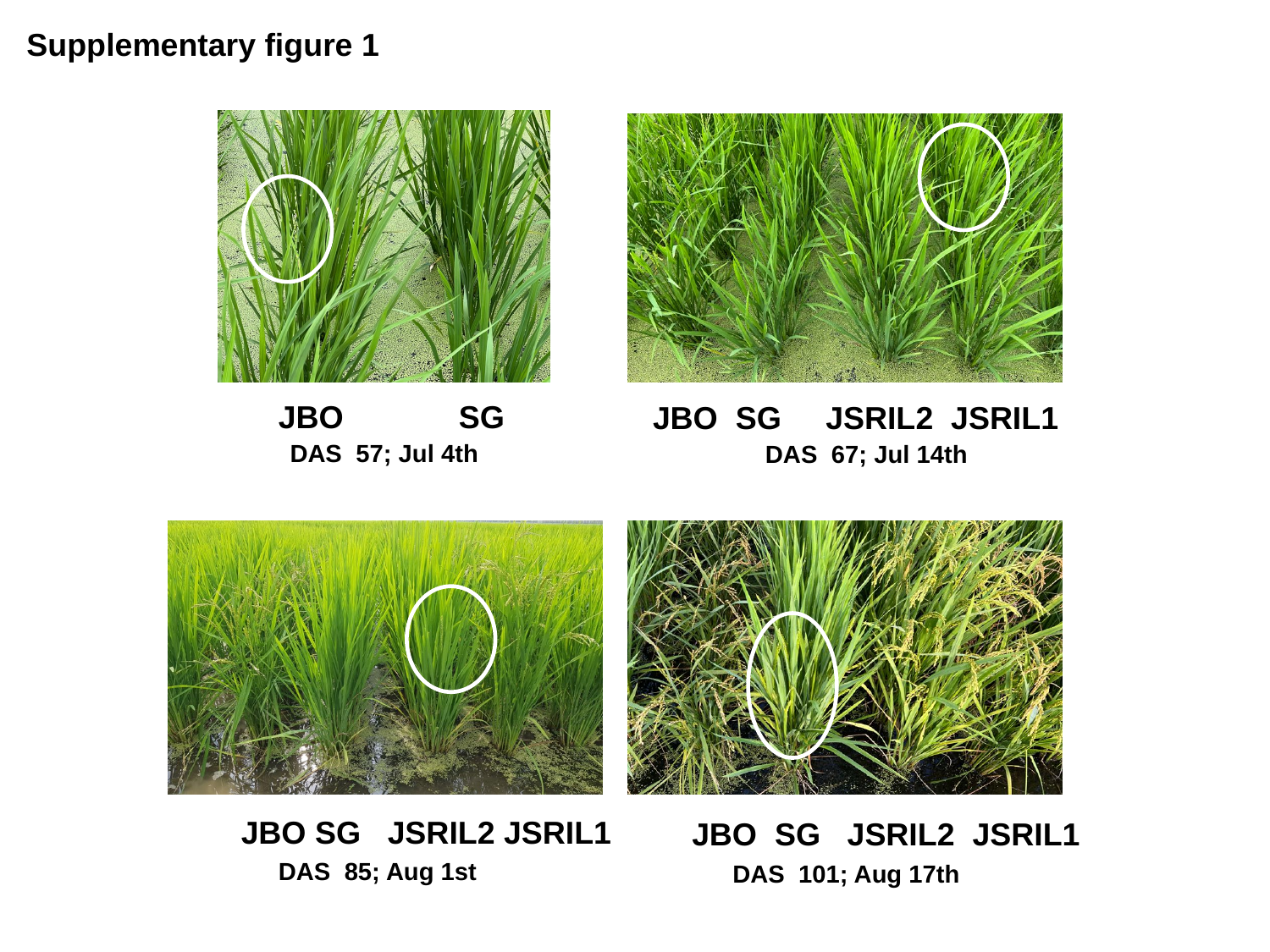

Supplementary figure 1
JBO SG
JBO SG JSRIL2 JSRIL1
DAS 57; Jul 4th
DAS 67; Jul 14th
JBO SG JSRIL2 JSRIL1
JBO SG JSRIL2 JSRIL1
DAS 85; Aug 1st
DAS 101; Aug 17th

## Slide 2
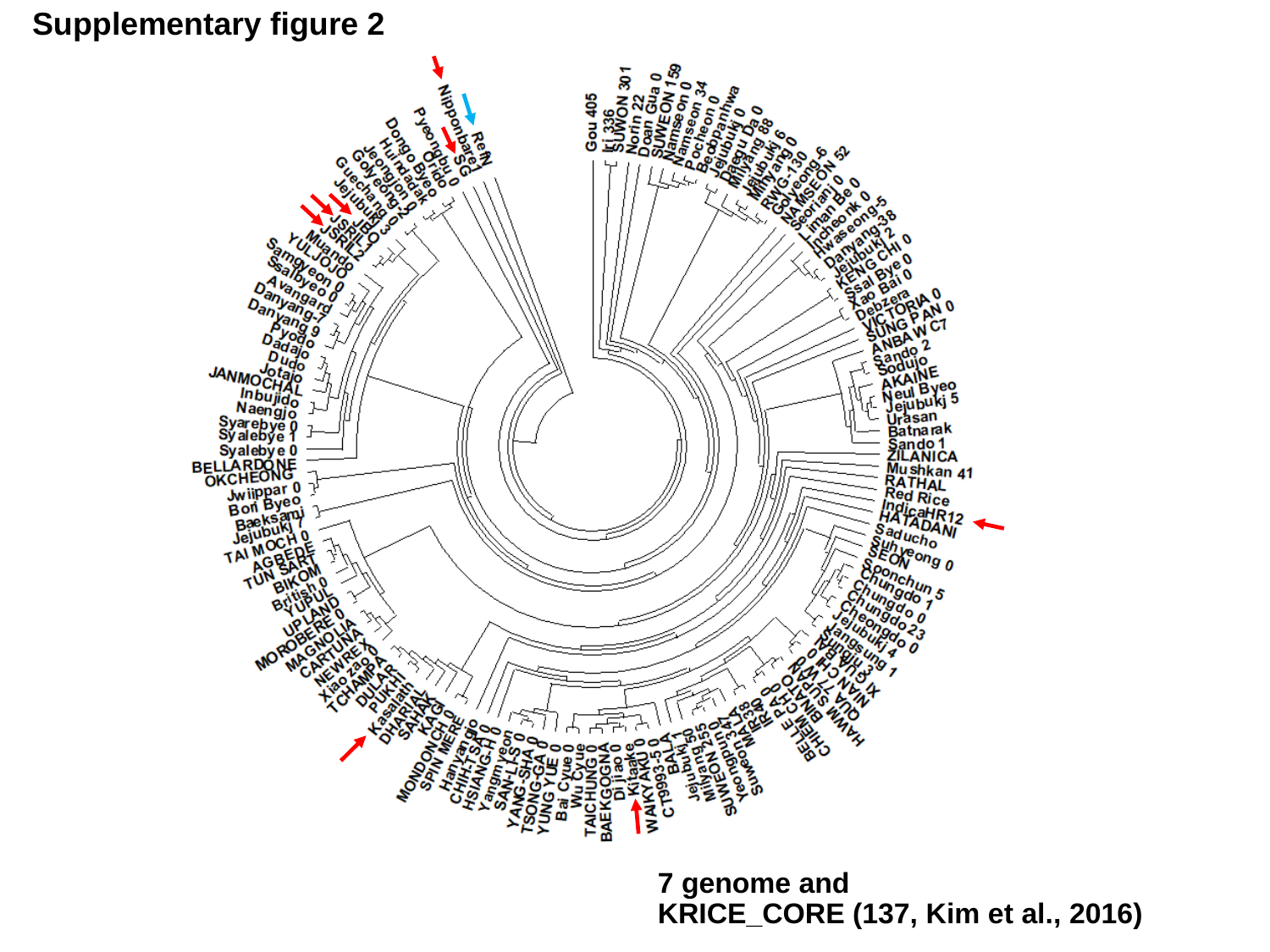

Supplementary figure 2
7 genome and
KRICE_CORE (137, Kim et al., 2016)

## Slide 3
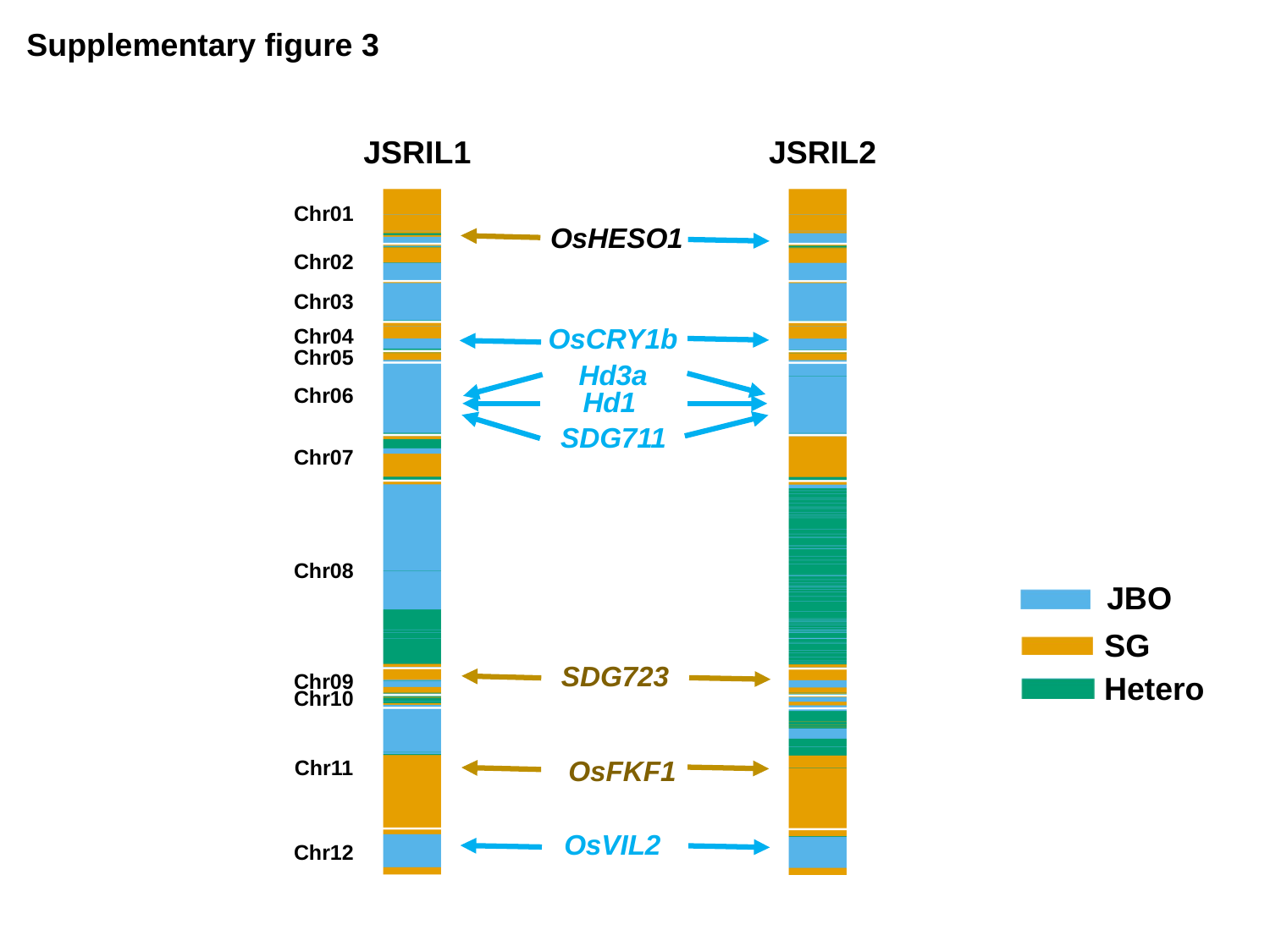

Supplementary figure 3
JSRIL1
JSRIL2
Chr01
OsHESO1
Chr02
Chr03
OsCRY1b
Chr04
Chr05
Hd3a
Chr06
Hd1
SDG711
Chr07
Chr08
JBO
SG
SDG723
Chr09
Hetero
Chr10
OsFKF1
Chr11
OsVIL2
Chr12

## Slide 4
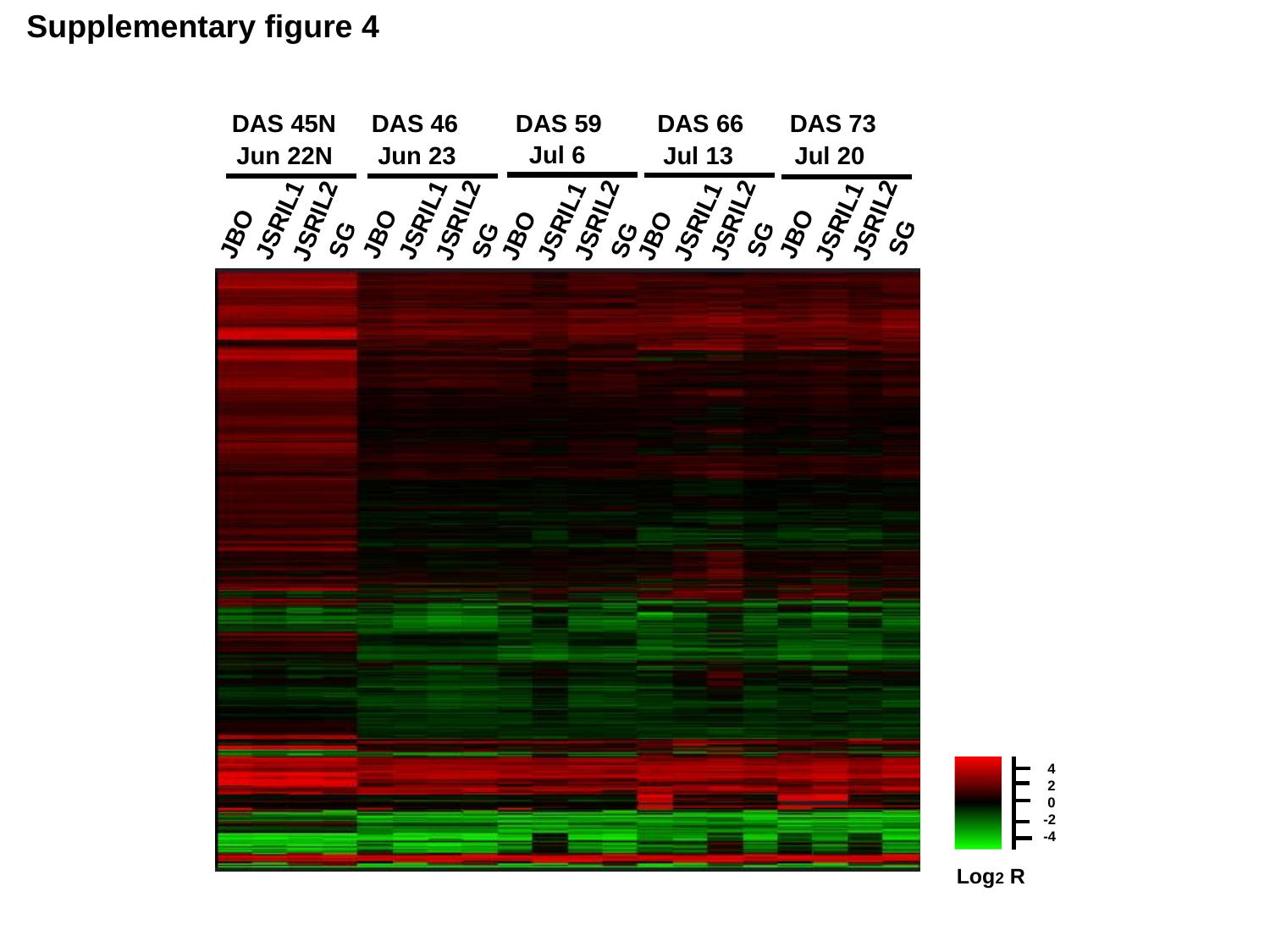

Supplementary figure 4
DAS 59
DAS 46
DAS 73
DAS 45N
DAS 66
Jul 6
Jun 23
Jul 20
Jun 22N
Jul 13
JSRIL1
JSRIL2
JSRIL1
JSRIL2
JSRIL2
JSRIL2
JSRIL2
JSRIL1
JSRIL1
JSRIL1
JBO
JBO
JBO
JBO
JBO
SG
SG
SG
SG
SG
 4
 2
 0
-2
-4
Log2 R
